# An externally validated clinical-laboratory nomogram for myocardial involvement in adult idiopathic-inflammatory-myopathy patients

**DOI:** 10.1007/s10067-024-06948-x

**Published:** 2024-04-08

**Authors:** Junyu Liang, Liyan Wan, Yake Yao, Xiao Cui, Ye He, Shuangshuang Li, Mengdi Jiang, Yiduo Sun, Heng Cao, Jin Lin

**Affiliations:** 1 Department of Rheumatology, the First Affiliated Hospital, Zhejiang University School of Medicine, #79 Qingchun Road, Zhejiang Province, Hangzhou, 310003 China; 2 Department of Respiratory Diseases, the First Affiliated Hospital, Zhejiang University School of Medicine, Hangzhou, China; 3 Department of Cardiology, the First Affiliated Hospital, Zhejiang University School of Medicine, Hangzhou, China

**Keywords:** Idiopathic inflammatory myopathy, Myocardial involvement, Anti-MDA5 antibody, Interleukin-17A

## Abstract

**Objectives:**

This study aimed at identifying clinical and laboratory risk factors for myocardial involvement (MI) in idiopathic inflammatory myopathies (IIMs) patients as well as constructing a risk-predicted nomogram for prediction and early identification of MI.

**Methods:**

An IIMs cohort in southeastern China was constructed, including 504 adult IIMs patients who met the inclusion and exclusion criteria, and were hospitalized at four divisions of the First Affiliated Hospital, Zhejiang University School of Medicine from January 1st 2018 to April 30st 2022. After dividing patients into the training cohort and the validation cohort, risk factors for MI were identified through least absolute shrinkage and selection operator regression and multivariate logistic regression. A risk-predicted nomogram was established and validated internally and externally for discrimination, calibration and practicability.

**Results:**

In this cohort, 17.7% of patients developed MI and the survival was significantly inferior to that of IIMs patients without MI (*P* < 0.001). In the training cohort, age > 55 years old (*P* < 0.001), disease activity > 10 points (*P* < 0.001), interleukin-17A (IL-17A) > 7.5 pg/ml (*P* < 0.001), lactic dehydrogenase (LDH) > 425 U/L (*P* < 0.001), anti-mitochondrial antibodies (AMAs, *P* = 0.017), and anti-MDA5 antibody (*P* = 0.037) were significantly correlated with development of MI. A nomogram was established by including the above values to predict MI and was found efficient in discrimination, calibration, and practicability through internal and external validation.

**Conclusion:**

This study developed and validated a nomogram model to predict the risk of MI in adult IIMs patients, which can benefit the prediction and early identification of MI as well as timely intervention in these patients.

**Supplementary Information:**

The online version contains supplementary material available at 10.1007/s10067-024-06948-x.

**Table:** 

**Key Points** *• Serum IL-17A was significantly correlated with myocardial involvement (MI) in idiopathic-inflammatory-myopathies (IIMs) patients.* *• Anti-MDA5-antibody-postive IIMs patients showed a remarkable tendency for complication of MI.* *• By including clinical and laboratory factors, the risk-predicted nomogram was valuable in predicting MI.*	

## Introduction

Idiopathic inflammatory myopathies (IIMs) are systemic inflammatory diseases with frequent damage to proximal skeletal muscles and skin, and partially unknown pathogenesis [1]. IIMs contribute to increased morbidity and mortality rate owing to severe muscle weakness as well as multiple organ involvement including esophagus, lungs, heart, hematological system, etc. [2, 3] In different cohorts, the mortality rate of IIMs approximately ranges from 10 to 30% [4–7]. The frequent multi-organ involvement and the unfavorable outcome have led to increasing researches seeking to figure out the pathophysiological mechanism, biomarkers, predictive models and therapeutic regimens for IIMs and their complications [8–10].

Previously interstitial lung disease (ILD) and its rapid progression (RP-ILD) has long occupied the summit in researches concerning IIMs and their different subgroups. However, myocardial involvement (MI) has gradually become another research focus in recent years. Due to different populations and diagnostic criteria, the incidence of MI in IIMs patients ranged from 5.5% to over 50%, which is frequently subclinical in its initial stage [11–13]. In preceding studies, multiple clinical and laboratory factors have been identified as risk factors for MI [13–15]. Besides, new imaging tools have been used for early identification [16, 17]. Despite the prosperity in MI researches, the pathophysiological mechanism, efficient biomarkers or predictive models remain unknown. Based on such background, a large IIMs cohort, which could reflect the IIMs spectrum in southeastern China, was established to probe into risk factors for MI in a more comprehensive manner and construct a risk-predicted nomogram model for MI in IIMs patients.

## Patients and methods

### Patients

To construct this IIMs cohort, we retrieved medical records and follow-up documents of adult patients who were hospitalized at Qingchun, Chengzhan, Yuhang and Zhijiang divisions of the First Affiliated Hospital, Zhejiang University School of Medicine (FAHZJU) with discharge diagnosis of dermatomyositis (DM), polymyositis (PM), amyopathic dermatomyositis (ADM), immune-related necrotizing myopathy (IMNM) as well as inclusion body myositis (IBM) from January 1st 2018 to April 30st 2022. The patients in FAHZJU were mostly from Zhejiang province as well as the adjacent Anhui, Jiangsu, Jiangxi, Fujian, Henan provinces, which could reflect the health condition and disease spectrum of southeastern China. The inclusion criteria were as follows: 1) age over 18 years old; 2) the definite/probable diagnosis of DM, PM, ADM, IMNM or IBM satisfied the 2017 ACR/EULAR classification criteria, as confirmed by two experienced rheumatologists (Heng Cao and Yiduo Sun) [18]; whereas exclusion criteria encompassed: 1) certified overlap syndromes with other connective tissue diseases (CTDs); 2) myopathy related to thyroid dysfunction, strenuous exercise, drug-induced myositis (i.e., Chinese herbal medicine, lamivudine), inherited metabolic disorders, etc.; 3) hospitalization for reasons unrelated to myositis and its complications such as fracture, pregnancy, cataract and acquired immunodeficiency syndrome, owing to insufficient medical information for this study; 4) loss to follow-up without death from any cause within three months after hospitalization. The research protocol was ratified by the Institutional Review Board (IRB) of FAHZJU (Reference Number: 2022–237) and complied with the Declaration of Helsinki*.* Written informed consent on utilizing and publishing clinical data was acquired from all the patients included at hospitalization. The included patients were divided into a training cohort and a validation cohort as per the time of hospitalization and reasonable sample size.

### Clinical assessments

Clinical records of all the enrolled patients were screened and collected using the electronic medical record (EMR) system of the four divisions of FAHZJU. Clinical, imaging, and laboratory data were extracted and subsequently analyzed. Survival data were acquired from the follow-up records in multiple divisions. The detailed follow-up protocol has been documented in preceding study [19]. The end of follow-up was defined as death from any cause, loss to follow-up, or closure of follow-up for the purpose of this study (October 31st, 2022).

Baseline disease activity assessment, laboratory or radiological detections were fulfilled within the first week of hospitalization. On-admission IIMs disease activity was routinely measured utilizing the Myositis Disease Activity Assessment Visual Analogue Scales (MYOACT) [19]. Identification of MI, subclinical MI included, was made by a cardiologist (Xiao Cui) based on myocardial injury with sustained (≥ three times) cardiac troponin I (cTnI) > the 99th-percentile upper reference limit in different divisions (0.016 ng/ml, 0.034 ng/ml or 0.060 ng/ml as per different assay kits), or significantly abnormal myocardial findings on echocardiography or cardiac magnetic resonance imaging (MRI) during hospitalization [20]. The included IIMs patients were thus divided into MI group and non-MI group (control group). Besides, identifications of MI were all established after multidimentional assessments. ILD and RP-ILD were screened and confirmed by experienced respiratory specialists (Yake Yao ang Bingjue Ye) using lung high-resolution computed tomography (HRCT) [21–23]. Identification of infection and classification of immunosuppressive regimens were described in supplementary file 1 and 2.

### Laboratory detections

To acquire peripheral lymphocyte subsets, cytokines, anti-mitochondrial antibodies (AMAs) as well as profiles of myositis-specific antibodies (MSAs) and myositis-associated antibodies (MAAs), serum samples were routinely drawn and detected within the first week of hospitalization. Peripheral lymphocyte subsets were quantified as percentages of CD3^+^CD4^+^, CD3^+^CD8^+^, CD3^−^ CD19^+^, CD3^−^CD16^+^CD56^+^ cells utilizing CD45-PE, CD3-PC5, CD4-FITC, CD8-PE, CD19-FITC and CD3-FITC-CD (16 + 56)-PE mouse anti-human fluorescence monoclonal antibodies (BD Bioscience) and the BD FACScanto™ II flow cytometer (Becton Dickinson, San Jose, CA, USA). Meanwhile serum levels of Interleulin-2 (IL-2), IL-4, IL-6, IL-10, IL-17A, tumor necrosis factor (TNF)-α and interferon (IFN)-γ were determined using the cytometric bead array (CBA) kit BD™ CBA Human Th1/Th2/TH17 Cytokine Kit (BD Biosciences, San Jose, CA, USA) and the flow cytometer (described above). Assessment of cytokine was carried out as per the manufacturer’s instructions. The lower and upper limits of cytokine detection were 0.10 pg/ml and 5000.00 pg/ml, respectively. The data were engendered in graphical and tabular format using FCAP Array™ software (BD Biosciences, San Jose, CA, USA). AMAs were assessed using line immunoassay and indirect immunofluorescence as per the manufacturer’s instruction (Euroimmun, Lubeck, Germany). The MSAs (anti-MDA5, anti-Jo-1, anti-PL-7, anti-PL-12, anti-EJ, anti-OJ, anti-KS, anti-HATyr, anti-TIF1γ, anti-SAE1, anti-NXP2, anti-Mi-2α, anti-Mi-2β, anti-HMGCR, anti-SRP and anti-cN1A) and MAAs (anti-Ro-52, anti-PM-Scl75, anti-PM-Scl100, anti-Ku) were measured by an immunoblotting assay (EUROLINE Myositis Research Profile, EUROIMMUN, Lübeck, Germany) and the ELISA (INOVA, Falls Church, Virginia, United States) according to the manufacturers’ protocols.

### Statistical analysis

Statistical analysis was performed utilizing Graphpad Prism 9.0 and R 3.6.1. The *P* values in comparisons were adjusted by false discovery rate (FDR) correction to minimize type I error. After calculating reasonable sample size, constructing the training cohort and the validation cohort, the univariate logistic regression with the least absolute shrinkage and selection operator (LASSO) regularization and the multivariate logistic regression analysis were utilized to identify risk factors for development of MI in the training cohort. A risk-predicted nomogram was established and evaluated. For assessment of discrimination and calibration capacity as well as clinical practicability, receiver operating characteristics (ROC) curve, calibration curve, Hosmer Lemeshow test, decision curve analysis (DCA) and clinical impact curve (CIC) were used in internal (with 500 bootstrap resamples) and external validation. Meanwhile comparison of ROCs of nomogram and other items were implemented using the Delong test. Survivals among different subgroups were evaluated by the Kaplan–Meier method with log-rank test. All tests were two-sided, and *P* < 0.05 was deemed statistically significant. The R packages used in this study were listed in supplementary file 3.

## Results

### Patient characteristics

A total of 504 adult IIMs patients were included into this study (Supplementary file 4), encompassing 308 with DM, 99 with PM, 58 with ADM, 37 with IMNM and two with IBM. Anti-MDA5 antibody (20.0%) was found the most common MSA in this IIMs cohort (Supplementary file 5A). One hundred sixty-one (31.9%) were males and the median age of all the patients included was 57.00 (49.00, 66.00) years old. One hundred twenty-seven patients (25.2%) died in follow-up and the medium follow-up time was 18.15 (7.91, 31.93) months. Among the 504 patients, 89 patients (17.7%) developed MI, and the 415 patients without MI constituted the control group. Among the 89 IIMs patients with MI, 32.6% was anti-MDA5 antibody positive. (Supplementary file 5B) IIMs patients complicated with MI were found to suffer from significantly worse survival (*P* < 0.001, Supplementary file 6), with 31.5% of MI patients deceased within 3 months.

### Distribution of different factors in IIMs patients with or without MI

**Fig. 1:**
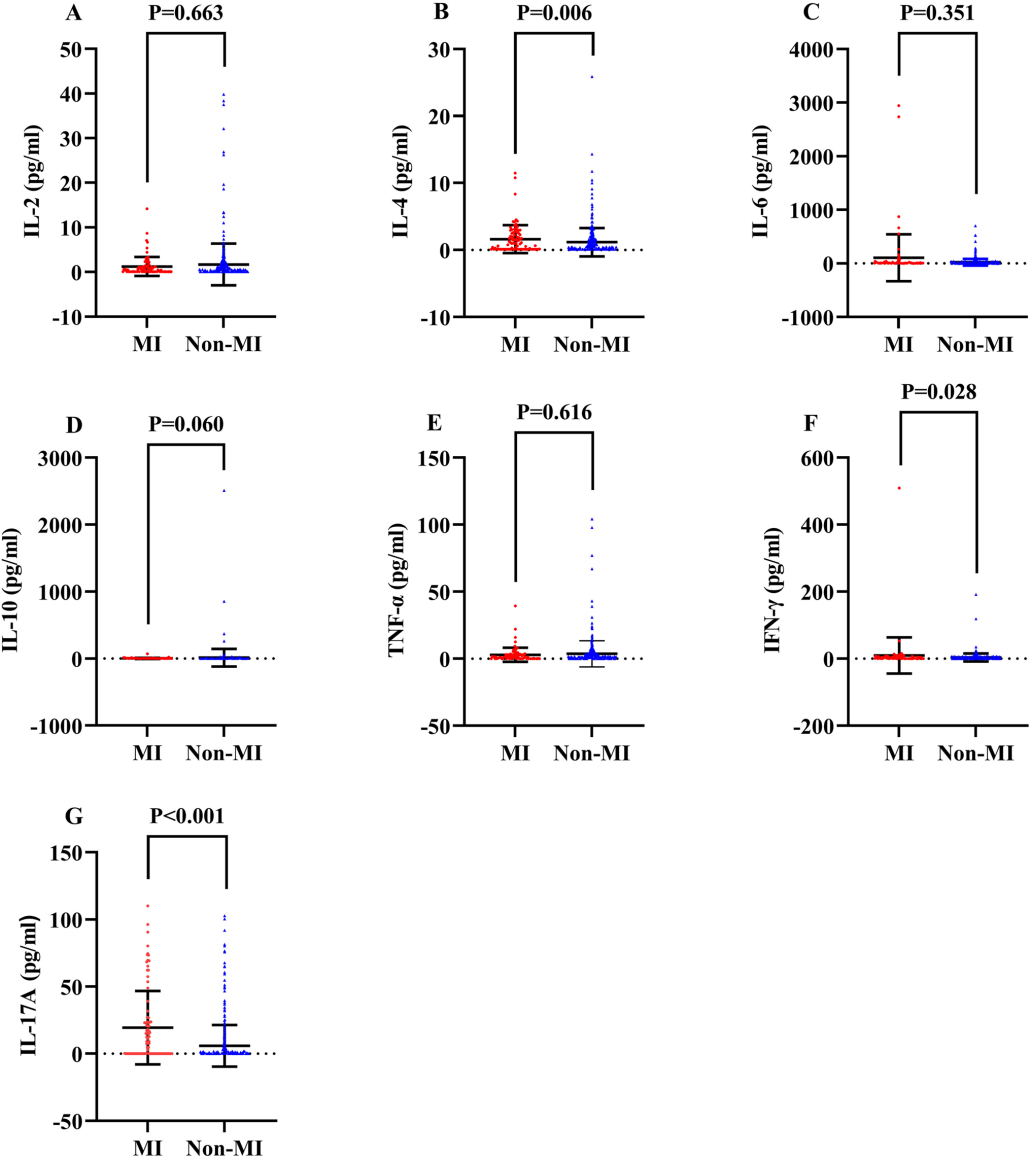
Comparisons of serum cytokines in IIMs patients with or without MI **A**. Comparison of serum IL-2 between MI and non-MI groups. **B**. Comparison of serum IL-4 between MI and non-MI groups. **C**. Comparison of serum IL-6 between MI and non-MI groups. D. Comparison of serum IL-10 between MI and non-MI groups. **E**. Comparison of serum TNF-α between MI and non-MI groups. **F**. Comparison of serum IFN-γ between MI and non-MI groups. **G**. Comparison of serum IL-17A between MI and non-MI groups. IIMs: Idiopathic inflammatory myopathies; MI: Myocardial involvement; IL: Interleukin; TNF: Tumor necrosis factor; IFN: Interferon

Comparison between MI and non-MI groups identified that patients who developed MI were comparably older (*P* < 0.001), had shorter course of disease (*P* = 0.006), more periungual erythema (*P* = 0.020) and pharyngeal muscle involvement (*P* < 0.001), more complications of bacterial infection (*P* = 0.004), fungal infection (*P* < 0.001), RP-ILD (*P* = 0.016), pulmonary hypertension (*P* < 0.001) and hypertension (*P* = 0.003), elevated MYOACT score (*P* < 0.001), lower percentage of peripheral CD3^+^CD4^+^ lymphocytes (*P* = 0.003), higher proportion of peripheral CD3^−^CD16^+^CD56^+^ lymphocytes (*P* = 0.029), higher serum levels of Interleukin-4 (IL-4, *P* = 0.006, Fig. 1B), IFN-γ (*P* = 0.028, Fig. 1F), IL-17A (*P* < 0.001, Fig. 1G), ferritin (*P* < 0.001), carcinoembryonic antigen (CEA, *P* = 0.001), CA125 (*P* = 0.014), CA199 (*P* = 0.001), lactate dehydrogenase (LDH, *P* < 0.001), creatine kinase (CK, *P* = 0.002), as well as positivity of AMAs (*P* = 0.010), anti-MDA5 antibody (*P* = 0.002), anti-Jo-1 antibody (*P* = 0.033), anti-NXP2 antibody (*P* = 0.013), anti-SRP antibody (*P* = 0.014), and anti-Ro52 antibody (*P* = 0.041), higher maximum dosage of steroid (*P* < 0.001), less usage of steroid + DMARDs (*P* = 0.048) and more application of steroid + DMARDs + IVIG (*P* = 0.002), less subtype of DM (*P* = 0.018).

After FDR correction, nevertheless, less clinical and laboratory factors remained statistically significant, encompassing older age (*P*-adjusted < 0.001), shorter course of disease (*P*-adjusted = 0.028), more pharyngeal muscle involvement (*P*-adjusted < 0.001), more complications of bacterial infection (*P*-adjusted = 0.021), fungal infection (*P*-adjusted < 0.001), pulmonary hypertension (*P*-adjusted < 0.001) and hypertension (*P*-adjusted = 0.017), elevated MYOACT score (*P*-adjusted < 0.001), lower percentage of peripheral CD3^+^CD4^+^ lymphocytes (*P* = 0.017), higher serum levels of IL-4 (*P*-adjusted = 0.028), IL-17A (*P*-adjusted < 0.001), ferritin (*P*-adjusted < 0.001), CEA (*P*-adjusted = 0.008), CA199 (*P*-adjusted = 0.008), LDH (*P*-adjusted < 0.001), and CK (*P*-adjusted = 0.013), as well as positivity of AMAs (*P*-adjusted = 0.045) and anti-MDA5 antibody (*P*-adjusted = 0.013), higher maximum dosage of steroid (*P*-adjusted < 0.001), more usage of steroid + DMARDs + IVIG (*P*-adjusted = 0.013) (Supplementary file 7).

### Establishment of the training cohort and the validation cohort

The minimum sample size required for new model development based on our study design was 276 patients with 47 events in terms of overall model fit (*R*^2^). According to the hospitalization time of patients, the 350 adult IIMs patients (with 63 MI) hospitalized from January 1st 2018 to January 31st 2021 constituted the training cohort, whereas the validation cohort was made up of the 154 adult IIMs patients (with 26 MI) hospitalized from February 1st 2021 to April 30st 2022. The ratio of sample size of the two cohorts was adjacent to 7:3.

### Establishment of the risk-predicted nomogram

**Fig. 2:**
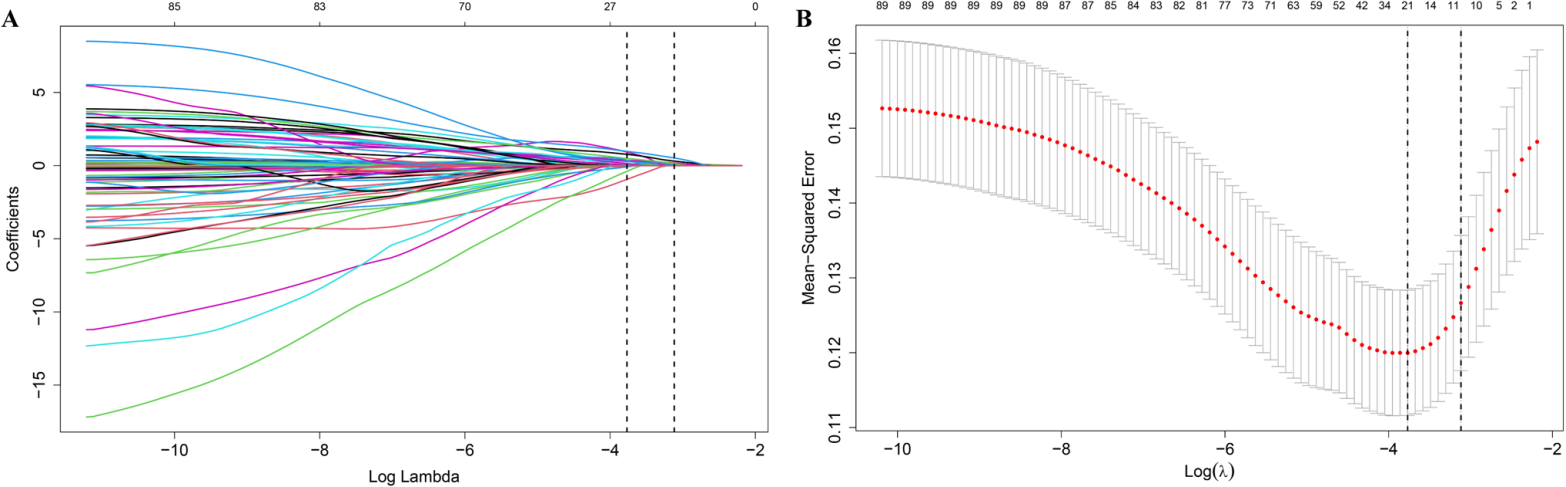
Selection of clinical or laboratory factors using logistic regression with the LASSO regularization. **A**. LASSO coefficient profiles of the clinical or laboratory factors. **B**. Tuning parameter (λ) selection in the LASSO model using 10-fold cross-testing via minimum criteria. LASSO: least absolute shrinkage and selection operator

**Fig. 3:**
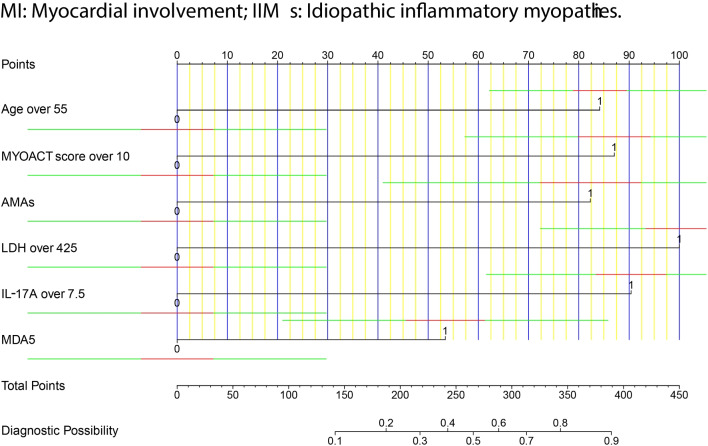
A risk-predicted nomogram for prediction of MI in IIMs patients. MI: Myocardial involvement; IIMs: Idiopathic inflammatory myopathies

Utilizing logistic regression with the LASSO regularization, the following 11 clinical or laboratory factors remained statistically significant in the training cohort: age, MYOACT score, hypertension, pharyngeal muscle involvement, pulmonary hypertension, fungal infection, AMAs, LDH, IL-6, IL-17A, anti-MDA5 antibody (Fig. 2A and 2B). To make the nomogram more practical in clinical practice, continuous variables including age, MYOACT score, LDH, IL-6 and IL-17A were rounded up and converted to binary variables based on ROC curve analyses (Supplementary file 8). The rounded-up cutoff values were over 55 years old, 10 points, 425 U/L, 2.5 pg/ml and 7.5 pg/ml, respectively. The 11 factors were afterwards enrolled into the multivariate logistic regression analysis. Age > 55 years old (*P* < 0.001), MYOACT score > 10 points (*P* < 0.001), AMAs (*P* = 0.017), LDH > 425 U/L (*P* < 0.001), IL-17A > 7.5 pg/ml (*P* < 0.001), and anti-MDA5 antibody (*P* = 0.037) were found to be significantly correlated with MI in adult IIMs patients (Table 1 and supplementary file 9). A risk-predicted nomogram incorporating the above six items were thus constructed (Fig. 3).

**Table 1:** Multivariate logistic regression analysis of MI among IIMs patients

Factors	*P* value	OR value	95% Confidence interval
Age (> 55y)	** < 0.001**	**4.455**	**1.992 ~ 9.963**
MYOACT score (> 10 points)	** < 0.001**	**4.686**	**2.254 ~ 9.743**
IL-17A (> 7.5 pg/ml)	** < 0.001**	**4.973**	**2.454 ~ 10.079**
LDH (> 425 U/L)	** < 0.001**	**5.898**	**2.885 ~ 12.058**
AMAs	**0.017**	**4.309**	**1.292 ~ 14.369**
MDA5	**0.037**	**2.581**	**1.059 ~ 6.287**

*MI* myocardial involvement; *IIMs* idiopathic inflammatory myopathies; *OR* odds ratio; *y* years; *MYOACT* Myositis Disease Activity Assessment Visual Analogue Scales; *IL* interleukin; *LDH* lactate dehydrogenase; *AMAs* anti-mitochondrial antibodies

### Internal and external validation of the risk-predicted nomogram

**Fig. 4:**
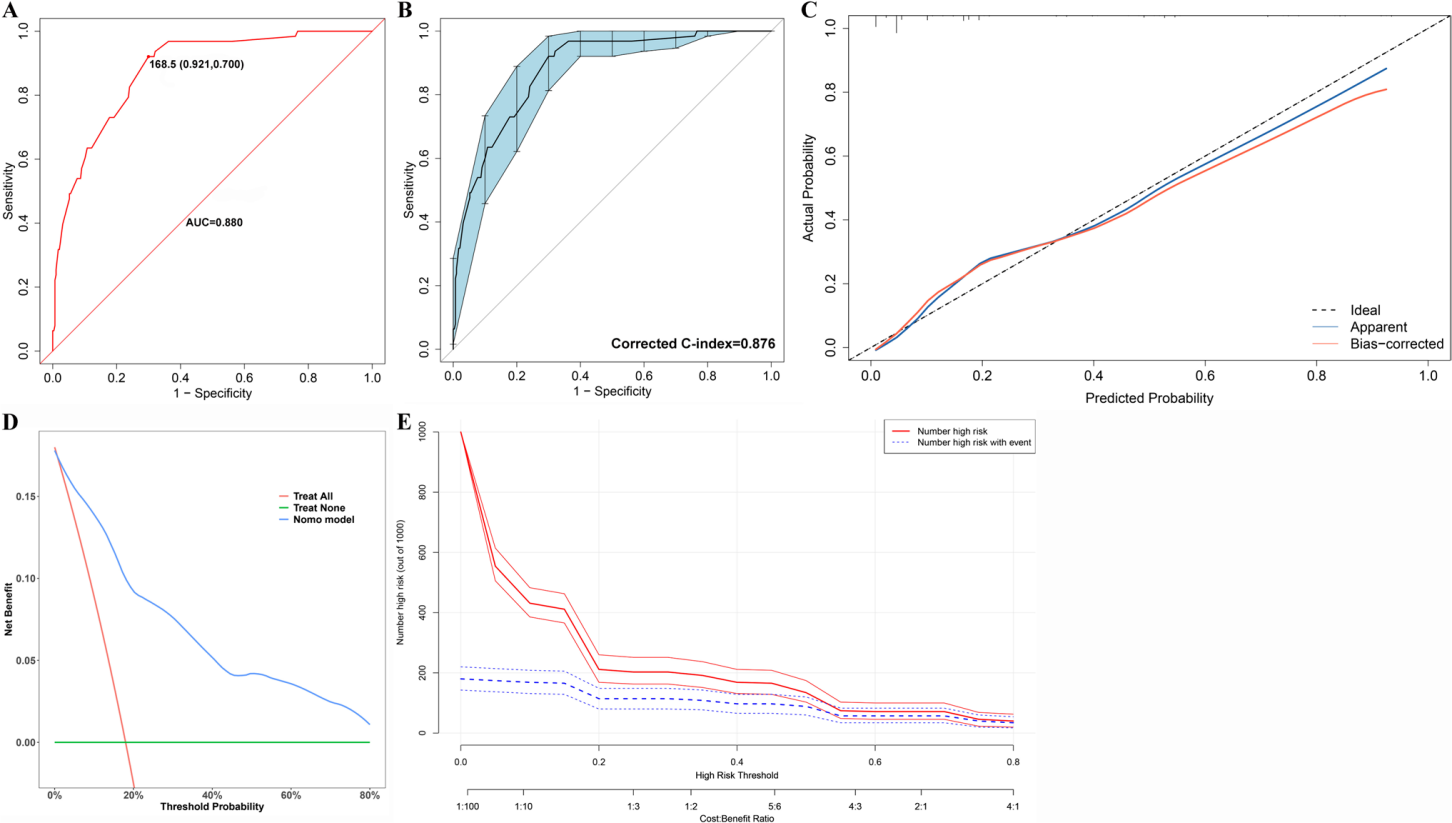
Internal validation of the nomogram. **A**. The ROC curve of the nomogram in training cohort; **B**. The ROC curve of the nomogram with 500 bootstrap resamples in the training cohort; **C**. The calibration curve of probability of MI for IIMs patients in training cohort. The x-axis and y-axis represented the nomogram-predicted probability and the actual probability of MI, respectively. Perfect prediction would correspond to the slope of black dotted line that made a 45° angle. The blue line represented the whole validation cohort (*n* = 350), and the red line indicated bias correction by bootstrap resamples (*B* = 500 repetitions), revealing the perfect agreement between MI and the risk prediction by the nomogram; **D**. DCA of the nomograms for development of MI in training cohorts. The x-axis represented the threshold probability, and the y-axis represents the net benefit calculated by adding the true positives and subtracting the false positives. The horizontal green line along the x-axis represented the assumption of IIMs patients without MI, whereas the diagonal red line represented the assumption that all IIMs patients developed MI at a specific threshold probability. The blue line represented the nomogram; **E**. CIC of the nomgram for the development of MI in the training cohort. The red line (i.e., number of high-risk patients) represented the number of patients classified as positive by the model at each threshold probability, meanwhile the blue curve (i.e., number of high-risk patients with an outcome) represented the number of patients with true positives at each threshold probability. ROC: Receiver operating curve characteristics; MI: Myocardial involvement; IIMs: Idiopathic inflammatory myopathies; DCA Decision curve analysis: CIC: Clinical impact curve

In the training cohort, the AUC of nomogram was 0.880 (95% CI 0.837–0.922), with cutoff value of ≥ 168.5, sensitivity of 92.1% and specificity of 70.0% (Fig. 4A). Meanwhile the bias-corrected C-index with 500 bootstraps was 0.876 (Fig. 4B), demonstrating excellent discrimination capacity. Besides, the calibration capacity of the nomogram was as well internally validated. The Hosmer–Lemeshow *p*-value of 0.758 in the training cohort suggested an excellent fitting of the nomogram. Furthermore, a Brier score of 0.098 and a calibration curve with 500 bootstrap resamples revealed satisfying agreement between the presence of MI and the risk prediction by the nomogram (Fig. 4C). In terms of the practicality of the model, the DCA of the risk-predicted nomogram revealed a superior overall net benefit for a threshold probability of 0–0.8 (Fig. 4D), and the CIC also identified satisfying performance over the entire range of threshold probabilities (Fig. 4E).

**Fig. 5:**
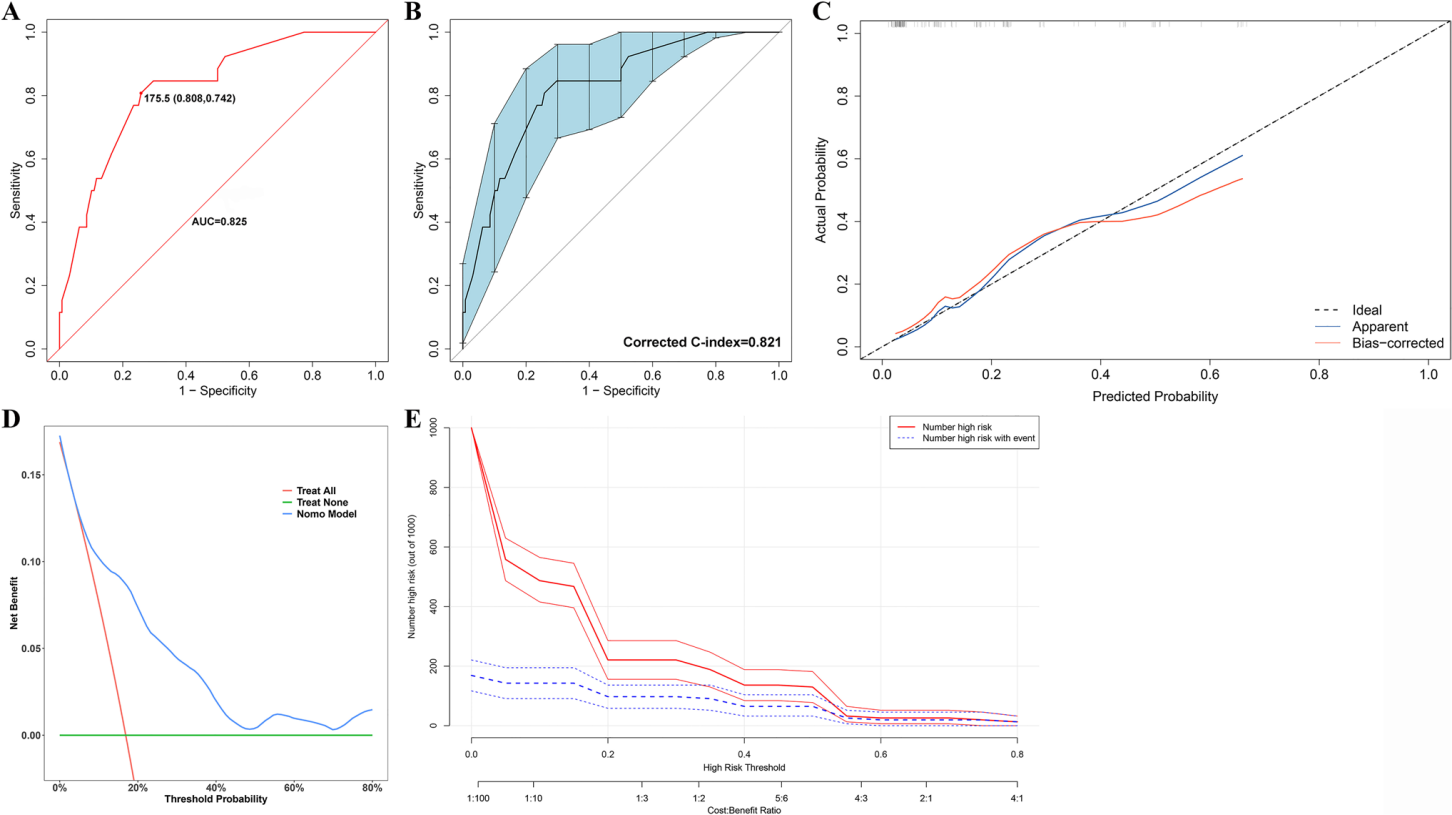
External validation of the risk-predicted nomogram. **A**. The ROC curve of the nomogram in validation cohort; **B**. The ROC curve of the nomogram with 500 bootstrap resamples in the validation cohort; **C**. The calibration curve of probability of MI for IIMs patients in validation cohort. The x-axis and y-axis represented the nomogram-predicted probability and the actual probability of MI, respectively. Perfect prediction would correspond to the slope of black dotted line that made a 45° angle. The blue line represented the whole validation cohort (*n* = 154), and the red line indicated bias correction by bootstrap resamples (*B* = 500 repetitions), revealing the perfect agreement between MI and the risk prediction by the nomogram; **D**. DCA of the nomograms for development of MI in validation cohorts. The x-axis represented the threshold probability, and the y-axis represents the net benefit calculated by adding the true positives and subtracting the false positives. The horizontal green line along the x-axis represented the assumption of IIMs patients without MI, whereas the diagonal red line represented the assumption that all IIMs patients developed MI at a specific threshold probability. The blue line represented the nomogram; **E**. CIC of the nomgram for the development of MI in the validation cohort. The red line (i.e., number of high-risk patients) represented the number of patients classified as positive by the model at each threshold probability, meanwhile the blue curve (i.e., number of high-risk patients with an outcome) represented the number of patients with true positives at each threshold probability. ROC: Receiver operating curve characteristics; MI: Myocardial involvement; IIMs: Idiopathic inflammatory myopathies; DCA Decision curve analysis: CIC: Clinical impact curve

In the validation cohort, the AUC of nomogram was 0.825 (95% CI 0.741–0.908), with cutoff value of ≥ 175.5, sensitivity of 80.8% and specificity of 74.2% (Fig. 5A). The bias-corrected C-index with 500 bootstraps was 0.821 (Fig. 5B), indicating consistent and satisfying discrimination capacity. In addition, the calibration capacity of the nomogram was also externally validated. The Hosmer–Lemeshow *p*-value of 0.420 in the validation cohort revealed an excellent fitting of the nomogram. Meanwhile a Brier score of 0.113 and a calibration curve with 500 bootstrap resamples indicated acceptable calibration of the nomogram model in the external validation cohort (Fig. 5C). Furthermore, The DCA and CIC were used to externally evaluate the clinical usefulness of our risk-predicted nomogram. The DCA showed a superior overall net benefit for a threshold probability of 0.1–0.8 (Fig. 5D). The CIC also exhibited satisfying performance over the entire range of threshold probabilities in the validation cohort (Fig. 5E).

Comparisons among the nomogram and the individual factors included in the model were carried out to further verify the discrimination and prediction ability of the nomogram. The AUC of the ROC curve of nomogram (AUC = 0.880) was prominently superior (*P* < 0.01, Delong test) to the AUCs of age > 55 years old (AUC = 0.647), MYOACT score > 10 points (AUC = 0.694), AMAs (AUC = 0.558), LDH > 425 U/L (AUC = 0.669), IL-17A > 7.5 pg/ml (AUC = 0.674) and anti-MDA5 antibody (AUC = 0.594) in the training cohort (Supplementary file 10A and 10B). Similar results could also be identified in the validation cohort (*P* < 0.05, Delong test) with AUCs of nomogram (AUC = 0.825), age > 55 years old (AUC = 0.530), MYOACT score > 10 points (AUC = 0.637), AMAs (AUC = 0.492), LDH > 425U/L (AUC = 0.733), IL-17A > 7.5 pg/ml (AUC = 0.634), and anti-MDA5 antibody (AUC = 0.533) (Supplementary Fig. 11A and 11B). Meanwhile, the DCA of the nomogram as well performed better than those of the individual factors in both the training cohort and the validation cohort (Supplementary file 10C, 10D, 11C and 11D).

Besides, to justify our action of transferring the continuous variables to binary variables, the ROC curves and DCAs of the transferred model (used in this study) or non-transferred model were as well compared. To be specific, the ROC curves of the two models showed similar (at least not inferior for the transferred model) discrimination capacity in both training cohort (AUC = 0.880 vs AUC = 0.859, P = 0.379, Delong test) and validation cohort (AUC = 0.825 vs AUC = 0.760, *P* = 0.188, Delong test) (Supplementary file 12A and 12B). The DCAs of the two models were neither found significantly different in the training cohort and the validation cohort (Supplementary file 12C and 12D).

## Discussion

Recent years have seen a prominent increase in clinical, imaging and laboratory studies concerning MI in IIMs patients. In this study, a large IIMs cohort in southeastern China, encompassing a large proportion of anti-MDA-antibody-positive patients, was analyzed to figure out risk factors as well as construct a risk-predicted nomogram for MI. IL-17A and anti-MDA5 antibody, together with other clinical factors, were found correlated with MI development. A risk-predicted nomogram consisting of both laboratory and clinical factors were thus constructed and validated (internally and externally) for prediction and early identification of MI in IIMs patients. Despite many of the MI events remain subclinical, IIMs patients with MI were found to suffer from more unfavorable outcome.

The IL-17 family of cytokines comprised of six proteins and five receptors, of which IL-17A and IL-17F are mostly produced by several types of immune cells [24]. The function of IL-17A has been expansively studied in different disease spectrum. In connective tissue diseases, IL-17A and IL-17A-producing cells have been demonstrated to influence focal inflammation, structural damage as well as new bone formation in psoriatic arthritis and ankylosing spondylitis [25]. Inhibition of IL-17A pathway via secukinumab, etc. has been used in treatment of these autoimmune diseases [26, 27]. However, the function of IL-17A in MI among CTD patients remains unknown. To date, this is the first study identifying the role of IL-17A in development of MI in IIMs patients. In myocardial diseases, IL-17A may play a role in cardiac fibrosis and remodeling, contributing to cardiomyopathy through modulating cardiomyocytes and cardiac fibroblast [28]. Apoptosis, autophagy, pyroptosis, neutrophil infiltration, etc. have been reported to be involved in these pathophysiological processes [29–31]. The detailed mechanisms behind IL-17A pathways in MI in IIMs patients, as well as efficacy of inhibiting IL-17A in repressing MI in these patients, demand further exploration.

IIMs patients with positivity of anti-MDA5 antibody has always been a research focus in the field of IIMs. By our observation, the frequency of anti-MDA5 antibody was comparably higher in different cohorts in southern China than those in northern China. The frequent development of RP-ILD, the scarce but fatal secondary hemophagocytic lymphohistiocytosis (sHLH), occurrence of opportunistic infection, involvement of pharyngeal and respiratory muscle, all contributed to the unfavorable outcome in these patients [15, 32–34]. Previously patients with positivity of anti-SRP antibody were reported to be vulnerable to cardiac involvement [35]. In 2022, Zeng, etc. reminded researchers and clinicians of the complication of MI in anti-MDA5 patients (15.8%) [15]. With more anti-MDA5 patients in our cohort, incidence of MI in anti-MDA5 patients was even higher (28.7%). In addition, positivity of anti-MDA5 antibody was also recognized as a risk factor for MI. In preceding studies, MDA5 seems to play a protective role in acute viral myocarditis since MDA5 is an RNA sensor that can induce transcription of type I IFN genes and thus defense against viruses [15]. However, in ischemic/reperfusion-injury and myocardial-infarction model, activation of type I IFN pathway aggravates myocardial injury [36, 37]. Whether MDA5 similarly activates type I interferon pathway and induce myocardial injury in IIMs patients demands further exploration.

The correlations between positivity of age, AMAs, LDH and MI have previously been reported [13–15, 38]. Elderly age was found associated with complication of carcinoma, infection, MI, ILD, etc. [13, 39, 40] In the rapidly aging society we are facing, IIMs patients with elderly age should be paid more attention to. In addition, positivity of AMAs was found associated with MI in IIMs patients, as well as in patients with myocarditis and idiopathic dilated cardiomyopathy, possibly owing to immune-related dysfunction of the cardiac cells by damage to the mitochondria [13, 14]. Furthermore, MYOACT score, as an overall but subjective disease activity assessment, was correlated with MI in IIMs patients. Similar phenomenon has been reported in critically ill patients with Corona Virus Disease 2019 (COVID-19) or community-acquired pneumonia [41]. The high disease activity may reflect high inflammatory burden which may subsequently induce MI [41].

Compared with merely identifying risk factors, establishment of a predictive model would not only assist in deepening our comprehensive understanding of IIMs and its complications, but also provide an efficient and instant tool for clinical practice. In preceding studies concerning adult or juvenile IIMs, various predictive models for prognosis, positivity of anti-MDA5 antibody, complication of hypertension, RP-ILD, sHLH and malignancy [19, 42–45] have been constructed. To date, this is the first study seeking to construct a comprehensive clinical model for prediction and early identification of MI in IIMs patients. On the basis of our study, we recommend a routine echocardiography and a low threshold for cardiac magnetic resonance imaging in patients with high risk, making detection of subclinical cardiac dysfunction early.

Several limitations existed in this study. Owing to our intention of focusing more on the clinical characteristics and peripheral serum biomarkers, as well as lack of cardiac MRI in a large proportion of MI patients, cardiac imaging factors were not included in this study. Although the patients in different divisions of FAHZJU reflect an overall condition in southeastern China, the cohort could only provide a regional (including multiple provinces), not national insight into IIMs. Also, this retrospective study included only hospitalized patients, which were the most complicated patients. This might limit the expansion of our nomogram for outpatient individuals.

## Conclusions

MI is a frequent complication in adult IIMs patients and contributes to unfavorable outcome. A nomogram encompassing IL-17A and anti-MDA5 antibody, together with age, disease activity, LDH and AMAs, was developed and validated (internally and externally) for prediction and early identification of MI in IIMs patients.

### Supplementary information

Below is the link to the electronic supplementary material.

Supplementary file1 (DOCX 20.3 KB)

Supplementary file2 (DOCX 20.2 KB)

Supplementary file3 (DOCX 22.5 KB)

Supplementary file4 (DOCX 3.76 MB)

Supplementary file5 (DOCX 116 KB)

Supplementary file6 (DOCX 129 KB)

Supplementary file7 (DOCX 40.1 KB)

Supplementary file8 (DOCX 204 KB)

Supplementary file9 (DOCX 118 KB)

Supplementary file10 (DOCX 7.60 MB)

Supplementary file11 (DOCX 7.23 MB)

Supplementary file12 (DOCX 6.82 MB)

Supplementary file13 (CSV 135 KB)

## Data Availability

The dataset supporting the conclusions of this article is provided in supplementary file 13.
