# Supplementary material for: An externally validated clinical-laboratory nomogram for myocardial involvement in adult idiopathic-inflammatory-myopathy patients
Source: Clin Rheumatol. 2024 Apr 8;43(6):1959–69. doi: 10.1007/s10067-024-06948-x (PMC11111495; doi:10.1007/s10067-024-06948-x)
Supplement: Supplementary file 1 — Supplementary file1 (DOCX 20.3 KB) [file 10067_2024_6948_MOESM1_ESM.docx]

**Supplementary file 1 Identification of different subtypes of infection**

EBV: Epstein-Barr virus; CMV: Cytomegalovirus; IgM: Immunoglobulin M; DNA: Deoxyribonucleic acid.

| Subtypes of infection | Identification strategy |
| --- | --- |
| bacterial, fungal, or tuberculosis  infection | A combined decision based on microbiological ﬁndings in sputum or blood, clinical manifestations, radiographic and laboratory abnormalities |
| Epstein-Barr virus (EBV) and cytomegalovirus (CMV) infection | Positive findings in the detection of serum IgM and DNA. |
