# Supplementary material for: An externally validated clinical-laboratory nomogram for myocardial involvement in adult idiopathic-inflammatory-myopathy patients
Source: Clin Rheumatol. 2024 Apr 8;43(6):1959–69. doi: 10.1007/s10067-024-06948-x (PMC11111495; doi:10.1007/s10067-024-06948-x)
Supplement: Supplementary file 2 — Supplementary file2 (DOCX 20.2 KB) [file 10067_2024_6948_MOESM2_ESM.docx]

**Supplementary file 2 Therapeutic regimens used in this cohort**

mPSL: Systemic methylprednisolone; PSL: Prednisolone; DMARDs: disease modifying anti-rheumatic drugs; JAK: Janus kinase; IVIG: intravenous immunoglobulin.

| Steroid monotherapy | Monotherapy of mPSL or PSL, the majority with a maximum dosage over 1 mg/kg/d (as calculated by PSL) |
| --- | --- |
| Combined therapy | combined therapy of PSL/mPSL as well as DMARDs or JAK inhibitors, with or without IVIG. |
| The DMARDs used for these patients encompassed Methotrexate, Cyclosporine, Tacrolimus, Mycophenolate, Thalidomide, Hydroxychloroquine and Cyclophosphamide. | |
| The JAK inhibitors applied in this cohort were Baricitinib and Tofacitinib. | |
