# Supplementary material for: An externally validated clinical-laboratory nomogram for myocardial involvement in adult idiopathic-inflammatory-myopathy patients
Source: Clin Rheumatol. 2024 Apr 8;43(6):1959–69. doi: 10.1007/s10067-024-06948-x (PMC11111495; doi:10.1007/s10067-024-06948-x)
Supplement: Supplementary file 3 — Supplementary file3 (DOCX 22.5 KB) [file 10067_2024_6948_MOESM3_ESM.docx]

**Supplementary file 3 Usage of different R packages in this study**

The “comparegroups” package was used for comparison between MI and non-MI patients. P values in comparisons were adjusted by false discovery rate (FDR) correction, utilizing “fdrtool” package. Calculation of reasonable sample size for the training cohort employed “pmsamplesize” package. Univariate logistic regression with the least absolute shrinkage and selection operator (LASSO) regularization was performed by the “glmnet” package to select significant factors that would be entered into multivariate logistic regression analysis. Using “glm” package and “forestmodel” package (for forest plot), the following multivariate logistic regression analysis was fulfilled to help establishing the risk-predicted nomogram. Establishment of the risk-predicted nomogram was implemented using the ‘rms’ package. For assessment of discrimination capacity, the Receiver operating characteristics (ROC) curves was employed. The ROC curves with or without 500 bootstrap resamples were plotted using the ‘pROC’ package. Meanwhile comparison of ROCs of nomogram and other items were carried out utilizing “ROCR” package. Differences in the AUC values between these models were analyzed using the Delong test. Evaluation of calibration capacity used the calibration curve. In detail, the calibration curve with 500 bootstrap resamples was plotted using “rms” package. The Hosmer Lemeshow test was performed using “HLtest” package. Decision curve analysis (DCA) and clinical impact curve (CIC) were applied for assessing practicality of the model. The “dcurves” package was utilized for DCA as well as comparisons of DCA. CIC was plotted using “rmda” package.
