# Supplementary material for: An externally validated clinical-laboratory nomogram for myocardial involvement in adult idiopathic-inflammatory-myopathy patients
Source: Clin Rheumatol. 2024 Apr 8;43(6):1959–69. doi: 10.1007/s10067-024-06948-x (PMC11111495; doi:10.1007/s10067-024-06948-x)
Supplement: Supplementary file 4 — Supplementary file4 (DOCX 3.76 MB) [file 10067_2024_6948_MOESM4_ESM.docx]

**Supplementary file 4 Enrollment and groupings of IIM patients**

IIM: Idiopathic inflammatory myopathy; MI: Myocardial involvement.

**
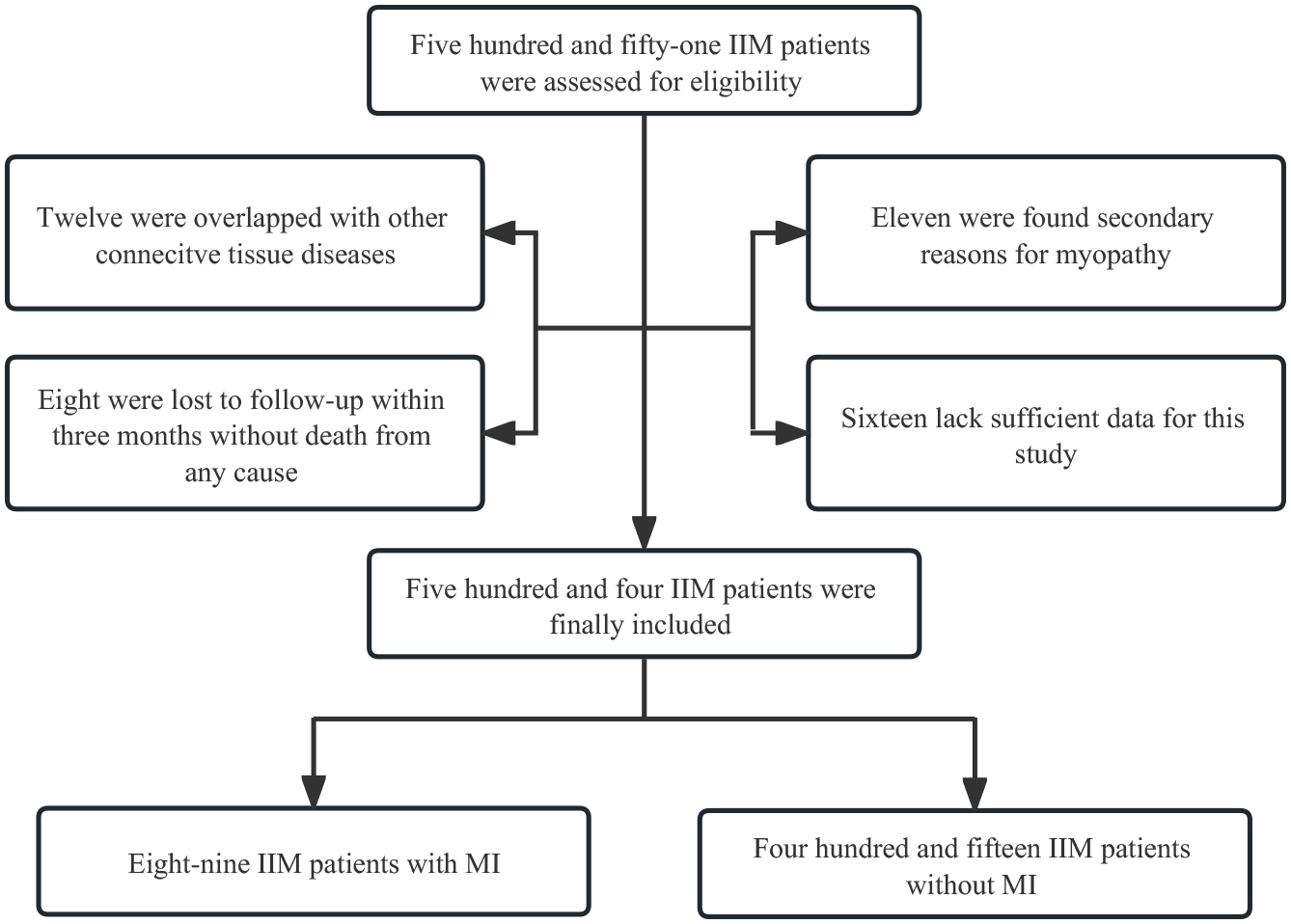
**
