# Supplementary material for: An externally validated clinical-laboratory nomogram for myocardial involvement in adult idiopathic-inflammatory-myopathy patients
Source: Clin Rheumatol. 2024 Apr 8;43(6):1959–69. doi: 10.1007/s10067-024-06948-x (PMC11111495; doi:10.1007/s10067-024-06948-x)

**Supplementary file 5 Distribution of anti-MDA5 antibody in IIM cohort as well as IIM patients with MI**

1. Distribution of anti-MDA5 antibody in IIM cohort.
2. Distribution of anti-MDA5 antibody in IIM patients with MI.

IIM: Idiopathic inflammatory myopathy; MI: Myocardial involvement.


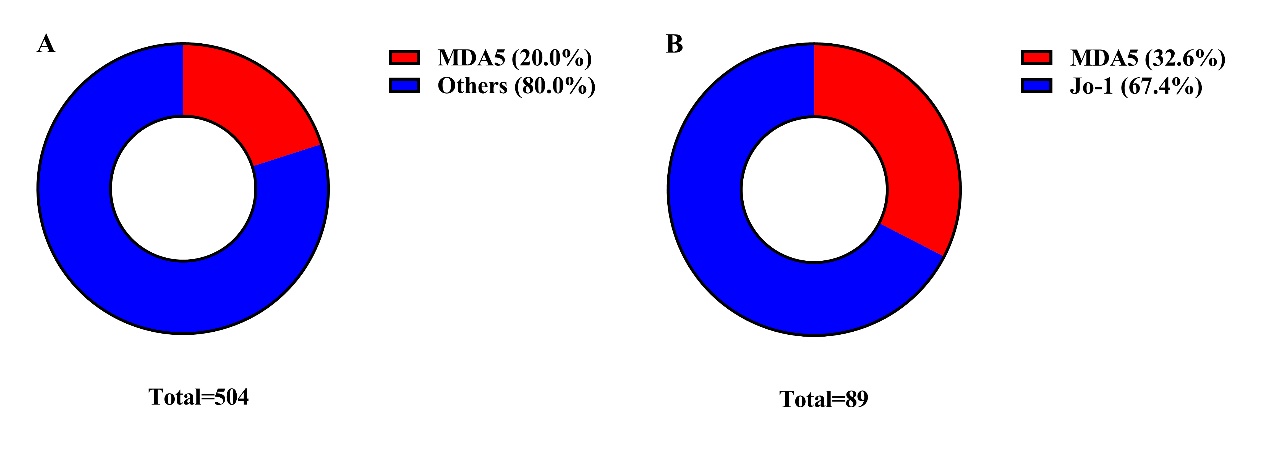

Supplement: Supplementary file 5 — Supplementary file5 (DOCX 116 KB) [file 10067_2024_6948_MOESM5_ESM.docx]
