# Supplementary material for: An externally validated clinical-laboratory nomogram for myocardial involvement in adult idiopathic-inflammatory-myopathy patients
Source: Clin Rheumatol. 2024 Apr 8;43(6):1959–69. doi: 10.1007/s10067-024-06948-x (PMC11111495; doi:10.1007/s10067-024-06948-x)
Supplement: Supplementary file 7 — Supplementary file7 (DOCX 40.1 KB) [file 10067_2024_6948_MOESM7_ESM.docx]

# Supplementary file 7 Comparisons of multiple factors between MI and non-MI groups

MI: Myocardial involvement; IIM: Idiopathic inflammatory myopathy; P-adjusted: Adjusted P value after false discovery rate correction; y: years; m: months; EBV: Epstein-Barr virus; CMV: Cytomegalovirus; ILD: Interstitial lung disease; RP-ILD: Rapidly progressive interstitial lung disease; MYOACT: Myositis Disease Activity Assessment Visual Analogue Scales; CD: Clusters of differentiation; IL: Interleukin; TNF: Tumor necrosis factor; IFN: Interferon; CRP: C-reactive protein; ESR: Erythrocyte sedimentation rate; LDH: Lactate dehydrogenase; CK: Creatine kinase; AMAs: Anti-mitochondrial antibodies; DMARDs*:* Disease-modifying anti-rheumatic drugs; IVIG: Intravenous immunoglobulin; JAK: Janus kinase; IIM: Idiopathic inflammatory myopathy; DM: dermatomyositis; PM: Polymyositis; ADM: Amyopathic dermatomyositis; IMNM: Immune-mediated necrotizing myopathy; IBM: Inclusion body myositis.

| **Factors** | **MI (89)** | **Non-MI (415)** | **P value** | **P-adjusted** |
| --- | --- | --- | --- | --- |
| **Age(y)** | **62.00(55.00,71.00)** | **56.00(47.00,65.00)** | **<0.001** | **<0.001** |
| **Sex(male/female)** | **30/59** | **131/284** | **0.694** | **0.950** |
| **Course of disease(m)** | **2.00(0.75,8.00)** | **3.00(1.00,12.00)** | **0.006** | **0.028** |
| **Duration of diagnosis delay(m)** | **1.00(0.50,5.00)** | **2.00(1.00,5.00)** | **0.175** | **0.360** |
| **Clinical manifestations or complications** | | | |  |
| **Heliotrope rash** | **29(32.6%)** | **155(37.3%)** | **0.468** | **0.731** |
| **Gottron’s sign** | **51(57.3%)** | **262(63.1%)** | **0.364** | **0.600** |
| **Periungual erythema** | **18(20.2%)** | **44(10.6%)** | **0.020** | **0.068** |
| **Mechanic’s hand** | **23(25.8%)** | **83(20.0%)** | **0.278** | **0.495** |
| **Raynaud sign** | **8(9.0%)** | **22(5.3%)** | **0.277** | **0.495** |
| **Pharyngeal muscle involvement** | **17(19.1%)** | **19(4.6%)** | **<0.001** | **<0.001** |
| **Articulator muscle involvement** | **3(3.4%)** | **14(3.4%)** | **1.000** | **1.000** |
| **Respiratory muscle involvement** | **3(3.4%)** | **6(1.5%)** | **0.201** | **0.398** |
| **Lymphadenectasis** | **32(36.0%)** | **179(43.1%)** | **0.260** | **0.482** |
| **Hepatomegaly** | **0(0.0%)** | **5(1.2%)** | **0.592** | **0.850** |
| **Splenomegaly** | **12(13.5%)** | **36(8.7%)** | **0.229** | **0.434** |
| **Bacterial infection** | **26(29.2%)** | **65(15.7%)** | **0.004** | **0.021** |
| **Fungal infection** | **20(22.5%)** | **35(8.4%)** | **<0.001** | **<0.001** |
| **Tuberculosis infection** | **2(2.3%)** | **9(2.2%)** | **1.000** | **1.000** |
| **EBV infection** | **18(20.2%)** | **52(12.5%)** | **0.083** | **0.205** |
| **CMV infection** | **7(7.9%)** | **12(2.9%)** | **0.058** | **0.161** |
| **ILD** | **77(86.5%)** | **350(84.3%)** | **0.722** | **0.959** |
| **RP-ILD** | **27(30.3%)** | **76(18.3%)** | **0.016** | **0.059** |
| **Pulmonary hypertension** | **12(13.5%)** | **13(3.1%)** | **<0.001** | **<0.001** |
| **Mediastinal emphysema** | **3(3.4%)** | **11(2.7%)** | **0.721** | **0.959** |
| **Gastrointestinal hemorrhage** | **7(7.9%)** | **18(4.3%)** | **0.178** | **0.360** |
| **Carcinoma** | **8(9.0%)** | **50(12.0%)** | **0.524** | **0.769** |
| **Preceding history** | | | | |
| **Smoking** | **17(19.1%)** | **72(17.3%)** | **0.810** | **0.979** |
| **Alcoholic abuse** | **14(15.7%)** | **68(16.4%)** | **1.000** | **1.000** |
| **Hypertension** | **33(37.1%)** | **90(21.7%)** | **0.003** | **0.017** |
| **Diabetes** | **14(15.7%)** | **47(11.3%)** | **0.329** | **0.574** |
| **Allergic history** | **14(15.7%)** | **51(12.3%)** | **0.481** | **0.738** |
| **Viral hepatitis** | **10(11.2%)** | **35(8.4%)** | **0.525** | **0.769** |
| **Disease activity** | | | |  |
| **MYOACT score** | **12.00(8.00,14.00)** | **8.00(6.00,10.00)** | **<0.001** | **<0.001** |
| **Laboratory findings** | | | |  |
| **CD3^+^CD4^+^ lymphocytes (%)** | **36.37±12.01** | **40.66±12.44** | **0.003** | **0.017** |
| **CD3^+^CD8^+^ lymphocytes (%)** | **27.59(17.01,35.46)** | **23.90(17.70,31.90)** | **0.363** | **0.600** |
| **CD4^+^/ CD8^+^ Ratio** | **1.34(0.86,2.46)** | **1.73(1.10,2.51)** | **0.086** | **0.207** |
| **CD3^-^CD16^+^CD56^+^ lymphocytes (%)** | **12.50(7.10,19.55)** | **9.99(6.00,16.40)** | **0.029** | **0.092** |
| **CD3^-^CD19^+^ lymphocytes (%)** | **16.30(9.20,24.30)** | **16.70(10.10,23.00)** | **0.812** | **0.979** |
| **IL-2 (pg/ml)** | **0.54(0.10,1.29)** | **0.39(0.10,1.40)** | **0.663** | **0.922** |
| **IL-4 (pg/ml)** | **1.00(0.10,2.47)** | **0.42(0.10,1.48)** | **0.006** | **0.028** |
| **IL-6 (pg/ml)** | **6.31(4.61,14.50)** | **6.65(3.06,19.70)** | **0.351** | **0.600** |
| **IL-10 (pg/ml)** | **3.34(2.04,4.58)** | **2.59(1.36,4.50)** | **0.060** | **0.162** |
| **TNF-α (pg/ml)** | **1.45(0.10,3.23)** | **1.27(0.10,3.43)** | **0.616** | **0.870** |
| **IFN-γ (pg/ml)** | **1.62(0.10,7.28)** | **0.94(0.10,4.15)** | **0.028** | **0.092** |
| **IL-17A (pg/ml)** | **7.39(0.10,23.00)** | **0.10(0.10.2.86)** | **<0.001** | **<0.001** |
| **CRP (mg/L)** | **5.70(3.00,23.40)** | **4.70(3.00,21.30)** | **0.772** | **0.968** |
| **ESR (mm/h)** | **19.00(8.00,35.00)** | **16.00(7.00,33.50)** | **0.762** | **0.968** |
| **Ferritin (ng/ml)** | **573.00(264.25,1539.80)** | **316.00(122.60,772.20)** | **<0.001** | **<0.001** |
| **Alpha-fetoprotein (ng/ml)** | **2.20(1.60,3.40)** | **2.30(1.61,3.30)** | **0.829** | **0.984** |
| **Carcinoembryonic antigen (ng/ml)** | **3.40(1.80,7.20)** | **2.40(1.40,3.85)** | **0.001** | **0.008** |
| **CA125 (U/ml)** | **19.90(12.50,40.40)** | **16.10(10.70,28.00)** | **0.014** | **0.054** |
| **CA199 (U/ml)** | **9.16(4.10,22.40)** | **5.30(2.60,12.90)** | **0.001** | **0.008** |
| **LDH (U/L)** | **476.00(331.00,619.00)** | **288.00(220.00,398.00)** | **<0.001** | **<0.001** |
| **CK (U/L)** | **391.00(62.00,1666.00)** | **158.00(51.00,736.00)** | **0.002** | **0.013** |
| **Positivity of AMAs** | **11(12.4%)** | **19(4.6%)** | **0.010** | **0.045** |
| **Myositis-specific antibodies & Myositis-associated antibodies** | | | |  |
| **Anti-MDA5** | **29(32.6%)** | **72(17.3%)** | **0.002** | **0.013** |
| **Anti-PL-7** | **8(9.0%)** | **45(10.8%)** | **0.744** | **0.968** |
| **Anti-PL-12** | **6(6.7%)** | **23(5.5%)** | **0.849** | **0.994** |
| **Anti-EJ** | **5(5.6%)** | **29(7.0%)** | **0.814** | **0.979** |
| **Anti-OJ** | **2(2.3%)** | **9(2.2%)** | **1.000** | **1.000** |
| **Anti-Jo-1** | **9(10.1%)** | **85(20.5%)** | **0.033** | **0.101** |
| **Anti-TIF1γ** | **3(3.4%)** | **35(8.4%)** | **0.156** | **0.356** |
| **Anti-Mi-2α** | **6(6.7%)** | **14(3.4%)** | **0.141** | **0.330** |
| **Anti-Mi-2β** | **7(7.9%)** | **18(4.3%)** | **0.178** | **0.360** |
| **Anti-SAE1** | **1(1.1%)** | **8(1.9%)** | **1.000** | **1.000** |
| **Anti-NXP2** | **0(0.0%)** | **25(6.0%)** | **0.013** | **0.054** |
| **Anti-SRP** | **10(11.2%)** | **16(3.9%)** | **0.014** | **0.054** |
| **HMGCR** | **1(1.1%)** | **8(1.9%)** | **1.000** | **1.000** |
| **Anti-Ku** | **2(2.3%)** | **13(3.1%)** | **1.000** | **1.000** |
| **Anti-PM-Scl75** | **2(2.3%)** | **16(3.9%)** | **0.752** | **0.968** |
| **Anti-PM-Scl100** | **1(1.1%)** | **7(1.7%)** | **1.000** | **1.000** |
| **Anti-Ro-52** | **39(43.8%)** | **234(56.4%)** | **0.041** | **0.122** |
| **KS** | **0(0.0%)** | **1(0.2%)** | **1.000** | **1.000** |
| **Hatyr** | **1(1.1%)** | **0(0.0%)** | **0.177** | **0.360** |
| **cN1A** | **0(0.0%)** | **2(0.5%)** | **1.000** | **1.000** |
| **Therapies** | | | |  |
| **Steroid monotherapy** | **15(16.9%)** | **85(20.5%)** | **0.527** | **0.769** |
| **Maximum dosage of steroid** | **100.00(75.00,200.00)** | **75.00(50.00,100.00)** | **<0.001** | **<0.001** |
| **Steroid+DMARDs** | **40(44.9%)** | **237(57.1%)** | **0.048** | **0.138** |
| [**Steroid+IVIG**](http://www.baidu.com/link?url=_srwKTXKnet8GknUvvs0xyTJdpfNOQtIDWHWhe_U5wypEldT9OPh2gCg3LsSDR-5CpyLTLOBAy4p4ov8wle8F6_YWPs4sPX-lyXINgDKaDW) | **12(13.5%)** | **34(8.2%)** | **0.171** | **0.360** |
| [**Steroid+DMARDs+IVIG**](http://www.baidu.com/link?url=uciYHxddnq2QF5VJVWJRCy7Q7nEAXlzzmiKvgGzZkrPg72XHW0qrc1acnFRmU-CtSPSZqd_rW-WBKuZFe0OpuS_h9gOsjyItDqvwfb_UtbdGjXJvU0FWCCPVF1qaXYLk) | **18(20.2%)** | **35(8.4%)** | **0.002** | **0.013** |
| **Steroid+JAK inhibitor** | **2(2.3%)** | **9(2.2%)** | **1.000** | **1.000** |
| **Steroid+DMARDs+JAK inhibitor** | **0(0.0%)** | **10(2.4%)** | **0.222** | **0.430** |
| **Steroid+IVIG+JAK inhibitor** | **2(2.3%)** | **1(0.2%)** | **0.082** | **0.205** |
| **Steroid+DMARDs+IVIG+JAK inhibitor** | **0(0.0%)** | **2(0.5%)** | **1.000** | **1.000** |
| **IIM subtypes** | | | |  |
| **DM** | **44(49.4%)** | **264(63.6%)** | **0.018** | **0.064** |
| **PM** | **21(23.6%)** | **78(18.8%)** | **0.375** | **0.607** |
| **ADM** | **13(14.6%)** | **45(10.8%)** | **0.409** | **0.650** |
| **IMNM** | **11(12.4%)** | **26(6.3%)** | **0.076** | **0.199** |
| **IBM** | **0(0.0%)** | **2(0.5%)** | **1.000** | **1.000** |
