# Supplementary material for: An externally validated clinical-laboratory nomogram for myocardial involvement in adult idiopathic-inflammatory-myopathy patients
Source: Clin Rheumatol. 2024 Apr 8;43(6):1959–69. doi: 10.1007/s10067-024-06948-x (PMC11111495; doi:10.1007/s10067-024-06948-x)
Supplement: Supplementary file 8 — Supplementary file8 (DOCX 204 KB) [file 10067_2024_6948_MOESM8_ESM.docx]

**Supplementary file 8 ROC curve of age, MYOACT score, LDH, IL-6 and IL-17A before transferring to binary variables**

A. ROC curve of age;

B. ROC curve of MYOACT score;

C. ROC curve of LDH;

D. ROC curve of IL-6;

E. ROC curve of IL-17A.

ROC: Receiver operating characteristics; MYOACT: Myositis Disease Activity Assessment Visual Analogue Scales; LDH: lactate dehydrogenase; IL: Interleukin.

**
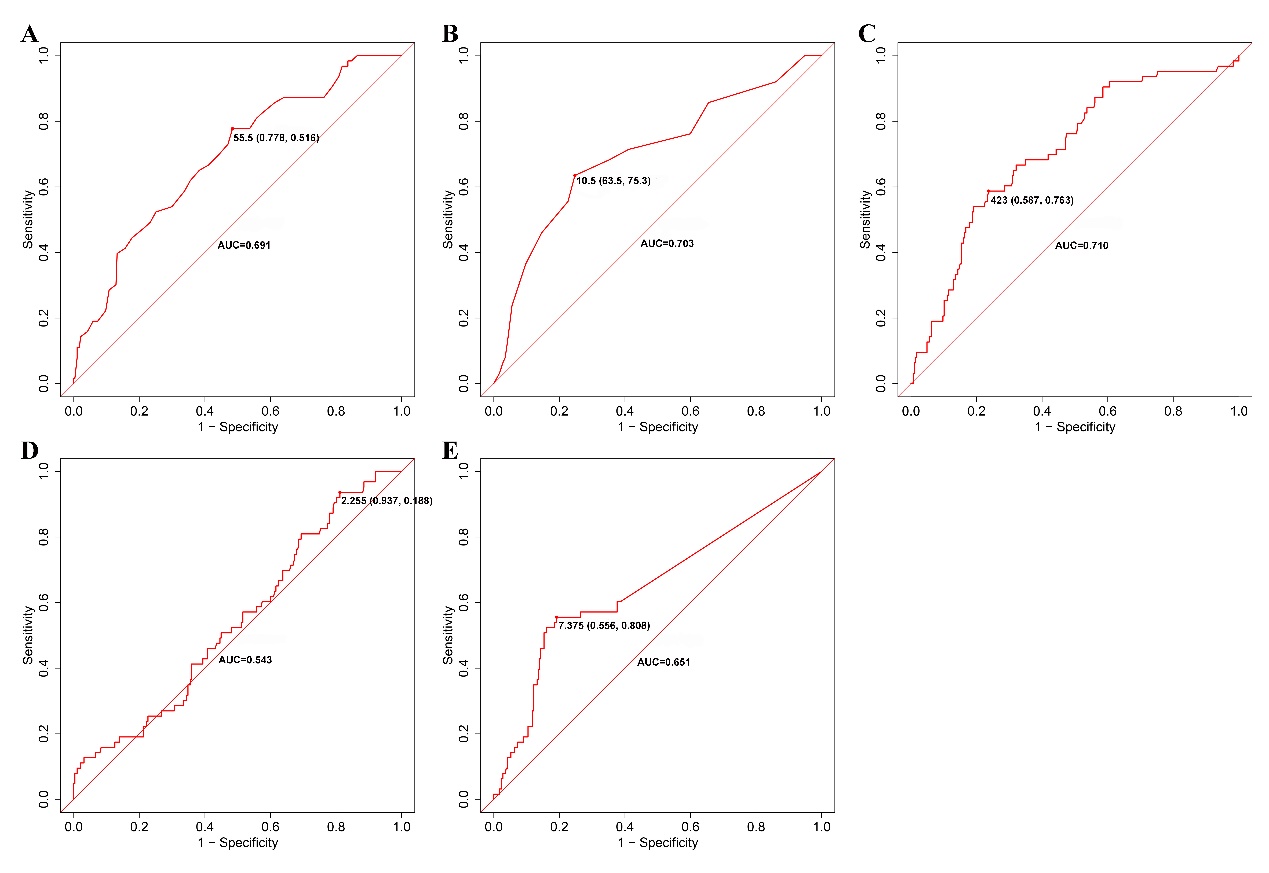
**
