# Supplementary material for: An externally validated clinical-laboratory nomogram for myocardial involvement in adult idiopathic-inflammatory-myopathy patients
Source: Clin Rheumatol. 2024 Apr 8;43(6):1959–69. doi: 10.1007/s10067-024-06948-x (PMC11111495; doi:10.1007/s10067-024-06948-x)
Supplement: Supplementary file 9 — Supplementary file9 (DOCX 118 KB) [file 10067_2024_6948_MOESM9_ESM.docx]

**Supplementary file 9 Forest plot for the multivariate logistic regression analysis**

MYOACT: Myositis Disease Activity Assessment Visual Analogue Scales; LDH: Lactate dehydrogenase; AMAs: Anti-mitochondrial antibodies; IL: Interleukin..

**
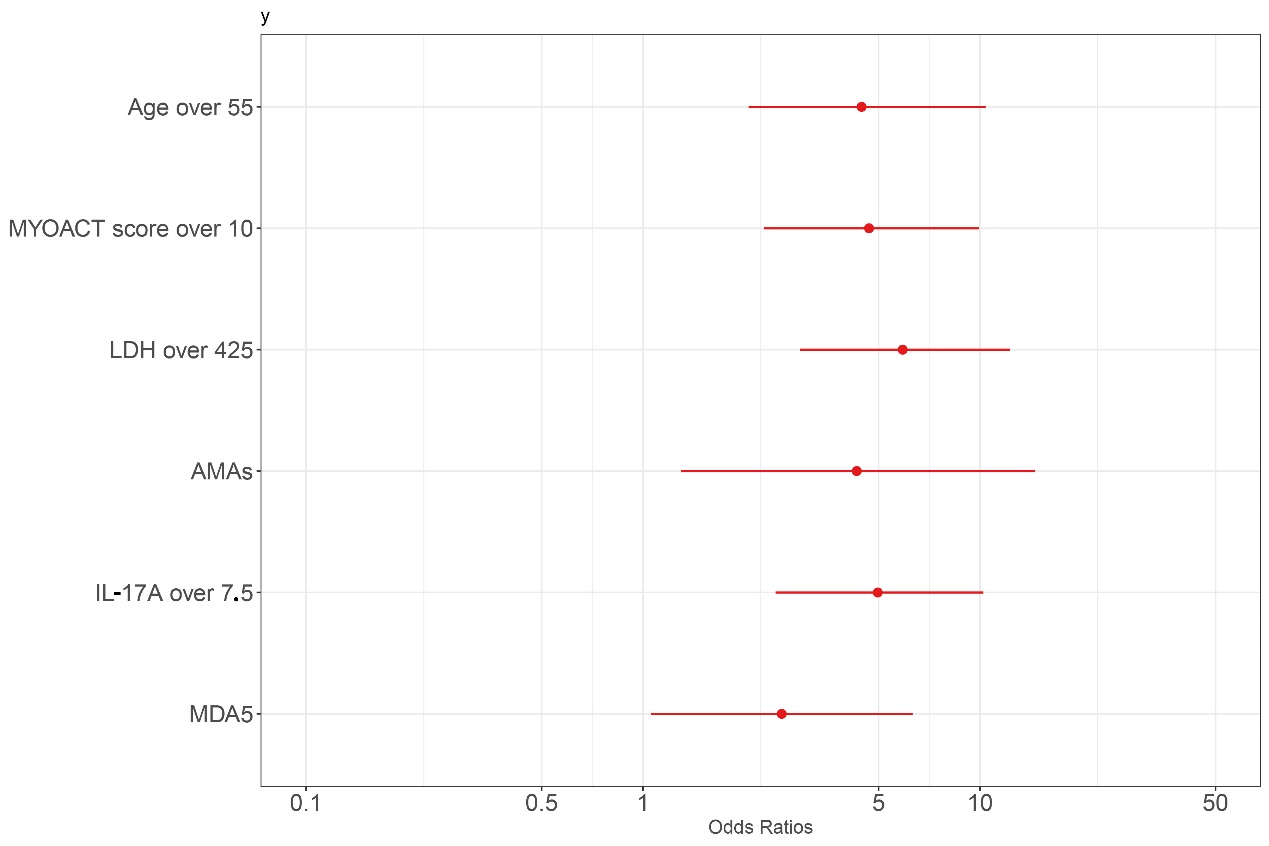
**
