# Supplementary material for: An externally validated clinical-laboratory nomogram for myocardial involvement in adult idiopathic-inflammatory-myopathy patients
Source: Clin Rheumatol. 2024 Apr 8;43(6):1959–69. doi: 10.1007/s10067-024-06948-x (PMC11111495; doi:10.1007/s10067-024-06948-x)

**Supplementary file 10 Comparisons of the risk-predicted nomogram and singular predictive factors in the training cohort**

A. ROCs of the nomogram, age> 55 years old, MYOACT score > 10 points and AMAs in the training cohort;

B. ROCs of the nomogram, LDH> 425 U/L, IL-17A > 7.5 pg/ml and anti-MDA5 antibody in the training cohort;

C. DCAs of the nomogram, age> 55 years old, MYOACT score > 10 points and AMAs in the training cohort;

D. DCAs of the nomogram, LDH> 425 U/L, IL-17A > 7.5 pg/ml and anti-MDA5 antibody in the training cohort.

ROC: Receiver operating characteristics; MYOACT: Myositis Disease Activity Assessment Visual Analogue Scales; AMAs: Anti-mitochondrial antibodies; LDH: lactate dehydrogenase; IL: Interleukin; DCA: Decision curve analysis.


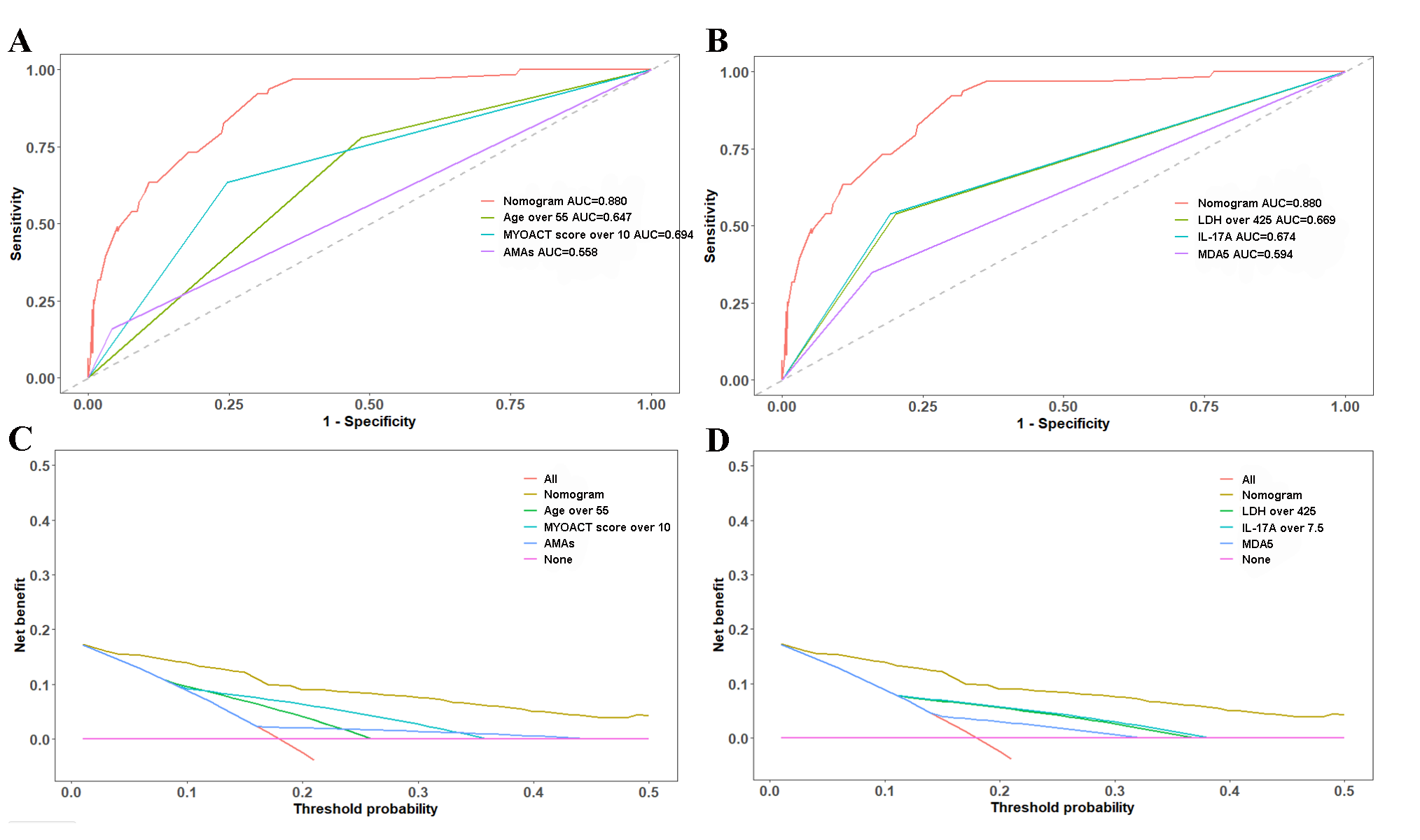

Supplement: Supplementary file 10 — Supplementary file10 (DOCX 7.60 MB) [file 10067_2024_6948_MOESM10_ESM.docx]
