# Supplementary material for: An externally validated clinical-laboratory nomogram for myocardial involvement in adult idiopathic-inflammatory-myopathy patients
Source: Clin Rheumatol. 2024 Apr 8;43(6):1959–69. doi: 10.1007/s10067-024-06948-x (PMC11111495; doi:10.1007/s10067-024-06948-x)

**Supplementary file 12 Comparisons of the transferred model and the non-transferred model in both the training cohort and the validation cohort**

A. ROCs of the transferred and the non-transferred model in the training cohort;

B. ROCs of the transferred and the non-transferred model in the validation cohort;

C. DCAs of the transferred and the non-transferred model in the training cohort;

D. DCAs of the transferred and the non-transferred model in the validation cohort;

ROC: Receiver operating characteristics; DCA: Decision curve analysis.


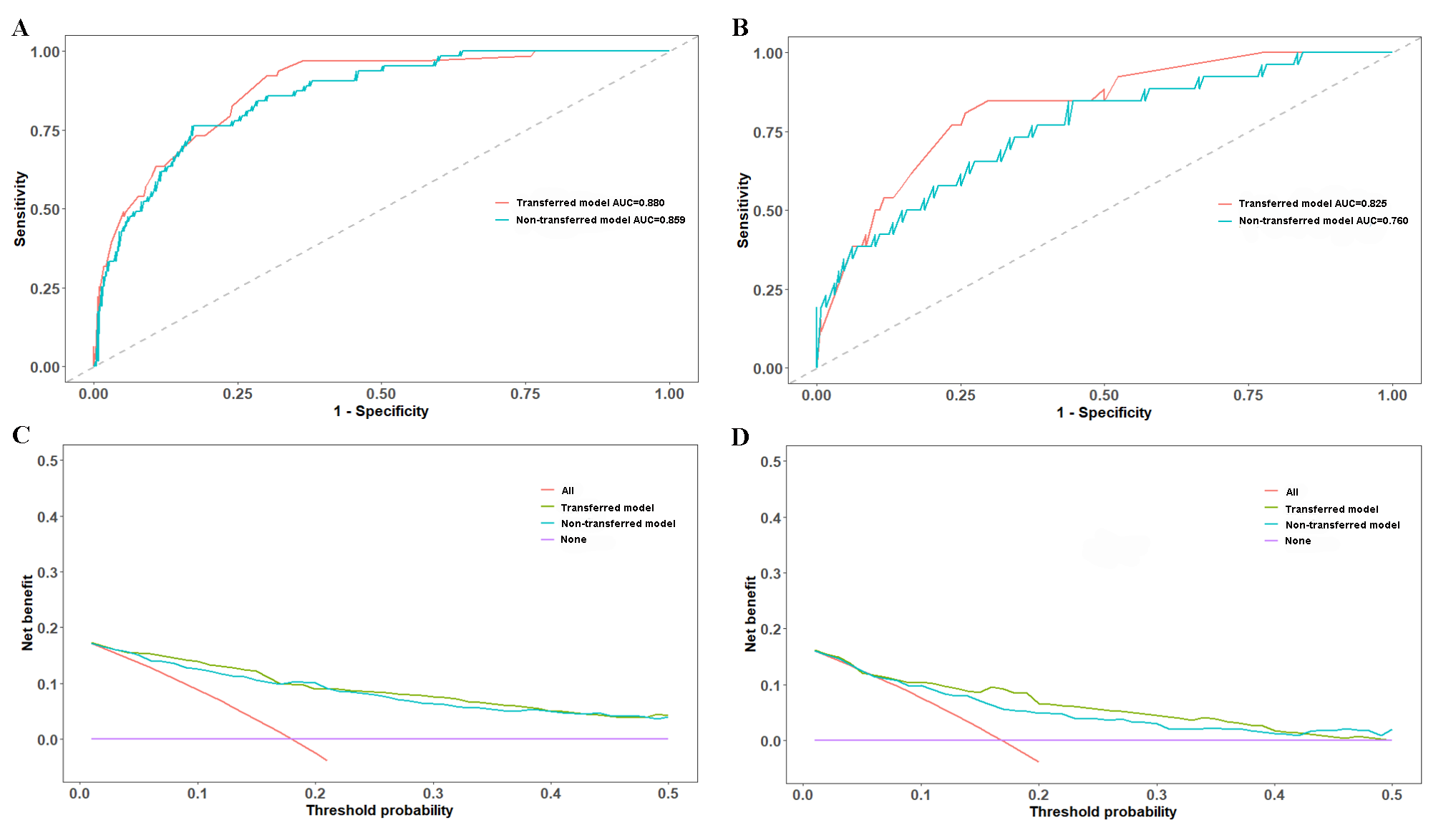

Supplement: Supplementary file 12 — Supplementary file12 (DOCX 6.82 MB) [file 10067_2024_6948_MOESM12_ESM.docx]
